# Major lithogenic contributions to the distribution and budget of iron in the North Pacific Ocean

**DOI:** 10.1038/s41598-019-48035-1

**Published:** 2019-08-12

**Authors:** Linjie Zheng, Yoshiki Sohrin

**Affiliations:** Institute for Chemical Research, Kyoto University, Gokasho, Uji, Kyoto 611-0011 Japan

**Keywords:** Marine chemistry, Element cycles

## Abstract

Recent studies have elucidated that iron (Fe) is a critical trace metal that influences the productivity of marine ecosystems and the biogeochemical cycles of other elements in the modern ocean. However, our understanding of the biogeochemistry of Fe remains incomplete. Herein, we report basin-scale and full-depth sectional distributions of total dissolvable iron (tdFe), dissolved iron (dFe), and labile particulate iron (lpFe = tdFe – dFe) in the North Pacific Ocean, as observed during three cruises of the GEOTRACES Japan program. We found that lpFe dominates tdFe and is significantly correlated with labile particulate aluminum (lpAl): lpFe [nmol kg^−1^] = (0.544 ± 0.005) lpAl [nmol kg^−1^] + 0.11 ± 0.04, *r*^2^ = 0.968, *n* = 432. The results indicate a major lithogenic contribution to the distribution of particulate Fe. For dFe, the unique distribution is attributed to the combined effects of biogeochemical cycling, manganese reduction, and lithogenic contribution. Based on concurrent observations of Fe, Al, and manganese (Mn), we infer that the width of the boundary scavenging zone is approximately 500 km off the Aleutian shelf. We estimate the inventory of tdFe in the North Pacific as 1.1 × 10^12^ mol, which is approximately four times that of dFe. Our results emphasize the potential importance of lpFe in the ocean’s iron cycle.

## Introduction

Although iron (Fe) is an abundant element in the upper crust, with an average concentration of 39 mg g^–1^ and a mole ratio of Fe/Al = 0.23^1^, it exhibits a low concentration in the order of nmol kg^−1^ in the modern ocean. This occurs because Fe forms Fe(III) hydroxide precipitates under the current oxic and neutral pH conditions and/or can be adsorbed onto particles. Martin *et al*. published vertical profiles of dissolved iron (dFe) in the ocean^2^, conducted bottle incubation experiments of phytoplankton with a controlled Fe concentration^3,4^, and subsequently proposed that Fe may be the limiting nutrient determining global rates of new production of phytoplankton in the modern ocean, as well as in the last glacial ocean^5^. The ‘iron hypothesis’ led to an increasing number of oceanographic studies of Fe, including mesoscale iron fertilization experiments in the open ocean^6–8^. Previous studies indicated that iron availability also controls nitrogen (N) fixation^9^, microbial phosphorus (P) acquisition^10^, and marine biogeochemical cycles of elements, including carbon (C) and sulfur (S)^11^. Additionally, it is suggested that nitrogen-iron co-limitation is pervasive in the ocean and affects phytoplankton community diversity^12^, and that the iron cycle exhibits feedback effects on climate and dust production^13^.

Many original studies and reviews have reported on the oceanic biogeochemical cycle of Fe^14,15^. However, the understanding of marine biogeochemistry of Fe remains incomplete, because Fe is incorporated in biogeochemical cycling in a manner similar to other nutrient elements and is also significantly influenced by scavenging throughout the water column and by redox reactions occurring at hydrothermal systems and within continental shelf sediments. The North Pacific Ocean occupies 21.3% of the world’s oceanic area and 24.8% of the world’s oceanic volume^16^, and is located at the end point of the thermohaline circulation of deep water. Specifically, the subarctic north-eastern Pacific and equatorial eastern Pacific are high-nutrient and low-chlorophyll regions^3–5^. Although several studies have examined the biogeochemistry of Fe in the North Pacific^17–24^, the following information is lacking. First, basin-scale sectional distributions and inventories of Fe are absent. Second, there is a scarcity of data on particulate iron (pFe) when compared to dFe. Most global marine biogeochemical iron cycling models do not focus on pFe^25,26^; however, pFe can be directly utilized by organisms^27–31^, and dFe–pFe interactions occur in seawater^32,33^. Third, there is a paucity of extensive studies on correlations between Fe and other trace metals, including aluminum (Al) and manganese (Mn), at the basin-scale, despite Al being proposed as a tracer of dust deposition of Fe to the ocean^34^, and Mn being a good tracer of redox cycling^35^.

The present study was conducted on samples and data acquired during the following cruises of R/V *Hakuho Maru*: KH-05-2 from August to September 2005, KH-11-7 in July 2011, and KH-12-4 from August to September 2012 (Supplementary Fig. 1). The KH-05-2 cruise represented a reconnaissance study for GEOTRACES Japan that covered a meridional section along 160°W. Additionally, on the KH-11-7 and KH-12-4 cruises, formal studies of GEOTRACES Japan were undertaken covering the GEOTRACES sections GP18 (165°E) and GP02 (47°N), respectively.

For the dissolved Fe analyses, a portion of seawater was passed through a filter with a pore size of 0.2 µm. Both filtered and unfiltered seawater samples were acidified with HCl to a final HCl concentration of 0.010 mol kg^–1^ (pH 2.2) and then stored at room temperature for at least 1 y until analysis. We used these samples to determine the dissolved (d) and total dissolvable (td) concentrations for Al, manganese (Mn), Fe, cobalt (Co), nickel (Ni), copper (Cu), zinc (Zn), cadmium (Cd), and lead (Pb)^36^. The difference between td and d concentrations is defined as the labile particulate (lp) concentration. The seawater data and their statistical summary are presented in Supplementary Tables 1 and 2, respectively. Previous studies^23,37^ reported on the local distributions of the nine metals around the Juan de Fuca Ridge and basin-scale distributions of Al, Mn, Co, and Pb in the North Pacific Ocean. Some of those data are included in the discussion section of the present study.

**Figure 1:**
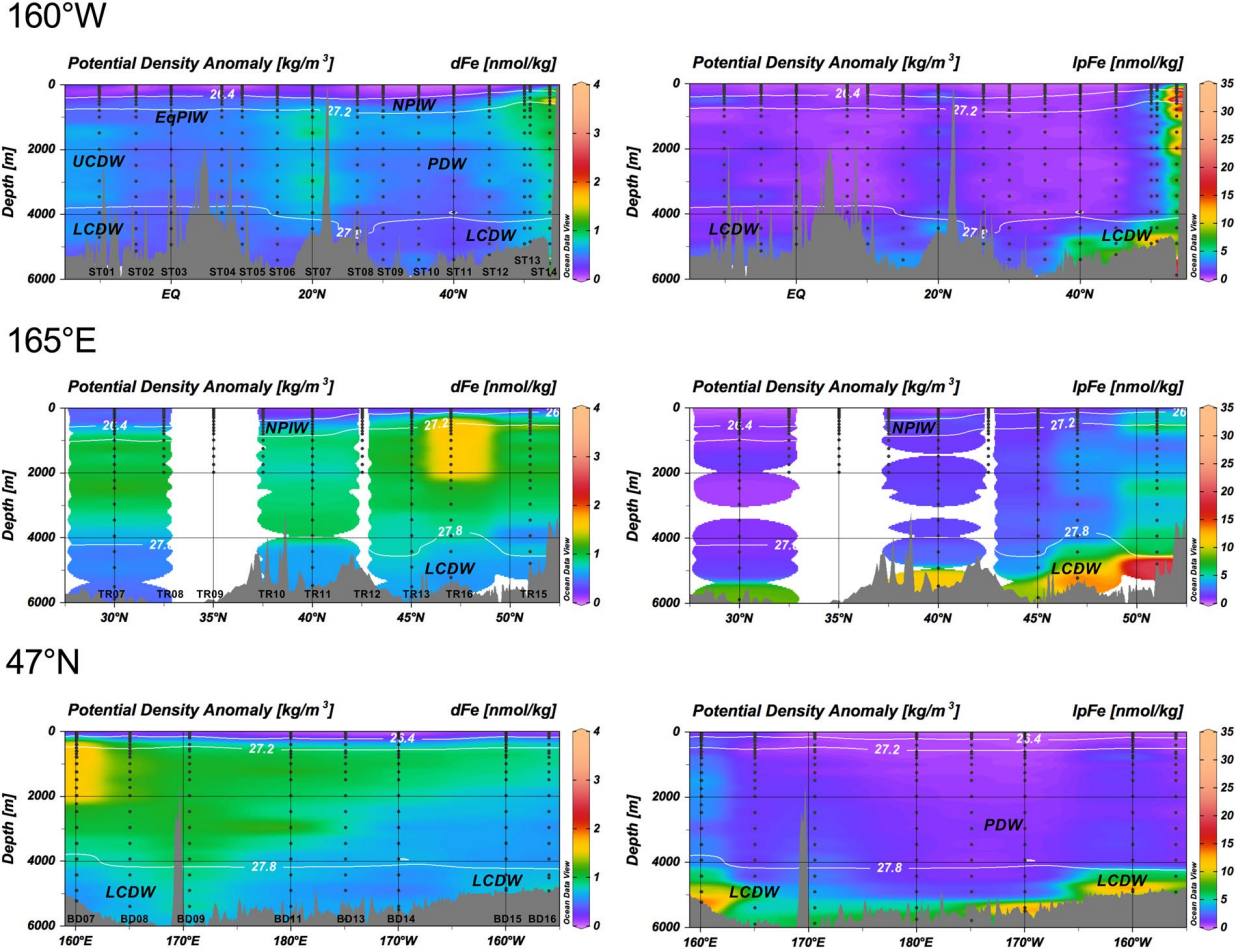
Full-depth sectional distribution of dFe and lpFe along 160°W, 165°E, and 47°N. The potential density isolines of 26.4 and 27.2 represent the upper and lower boundaries of the North Pacific Intermediate Water (NPIW), and those of 27.8 represent the upper boundary of the Lower Circumpolar Deep Water (LCDW). EqPIW: Equatorial Pacific Intermediate Water; UCDW: Upper Circumpolar Deep Water; PDW: Pacific Deep Water.

Figure 1 shows the full-depth sectional distributions of dFe and lpFe along 160°W, 165°E, and 47°N. Supplementary Figs 2 and 3 show the same distributions in the depth range of 0–1000 m and the sectional distributions of tdFe, respectively. The dFe was generally depleted in surface water, with the exception of stations in the Aleutian Stream (AS; ST14; 160.00°W, 53.58°N) and near the Hawaiian Islands (ST07; 160.002°N, 20.001°N). In deep water, the distribution of dFe illustrated the supply from the continental shelf and slope of the Aleutian Islands, the Sea of Okhotsk, and the Hawaiian Island Chains.

A sectional study of dFe along 158°W reported that two deep water anomalies were observed at depths of 1000–1500 m near the Hawaiian Islands and of 2000–3000 m in tropical South Pacific waters (7°S)^38^. The authors hypothesized that the anomalies were the result of hydrothermal Fe sources from the Loihi Seamount and the East Pacific Rise, based on coincidence with δ^3^He. In the present study, however, a hydrothermal tracer dMn did not exhibit the corresponding deep anomalies with dFe^37^. Thus, it is possible that a sediment source associated with the Hawaiian Island Chain was a more significant factor determining dFe during our observation.

The highest concentration of dFe (3.87 nmol kg^–1^) was observed at a depth of 149 m in the Aleutian Stream (AS). Lippiatt *et al*.^39^ observed high concentrations of reactive Fe in Alaska coastal waters and suggested that Fe originates from glacial rivers and streams and that dFe is maintained in seawater by Fe(III)-binding organic ligands.

The lpFe exhibited a distribution similar to that of lpAl^37^. High concentrations of lpFe were observed along the flow of the Lower Circumpolar Deep Water (LCDW), which enters the Central Pacific Basin through the Samoan Passage, proceeds further north, and reaches the Northeast Pacific Basin^40^ (Supplementary Fig. 4). The major branch flows anti-cyclonically along the Japan, Kuril–Kamchatka, and Aleutian Trenches. The LCDW finally upwells and is transformed into the Pacific Deep Water (PDW), which is internally formed entirely in the Pacific from the upwelling and diffusion of the LCDW. It is likely that lpFe and lpAl are transported via LCDW at a basin-scale and supplied locally by resuspension of sediments. The concentration of lpFe was also high near the continental shelf and slope. Off the Aleutian Islands, lpFe decreased sharply with respect to distance from the shelf break. The concentrations of tdFe, dFe, and lpFe in each water mass are compiled in Supplementary Table 3.

The concentrations of lpFe from ST09 (30.007°N, 159.996°W) were comparable with those of total pFe that correspond to the sum of acetic acid leachate and residual refractory fractions from the Vertex IV site (28°N, 155°W)^18^ (Supplementary Fig. 5). It is likely that the long-term acidic leach used in our study was recovering most of pFe: there was very little material that was refractory to our method. Similar results were obtained for lpAl and lpMn^37^. Around the Juan de Fuca Ridge, the lpM/lpAl ratios, where M stands for a metal element, in bottom water were close to the ratios of the total concentrations in the bottom sediments^23^. Additionally, the preservation period of the samples did not significantly affect the determination of tdM and dM in this study (see Methods). We conclude that both the unfiltered and filtered seawater samples at pH 2.2 were stable for the determination of tdM and dM from 1 to 10 y. Based on our results, we presume that lpM represent a major portion of total pM at open ocean stations. Berger *et al*. used the term “labile particulate” to represent a bioavailable fraction in particles^41^. They collected particles on filters with 10 µm and 0.4 µm pore sizes, and leached metals from the particles in 25% acetic acid with a mild reducing agent (0.02 M hydroxylamine hydrochloride) and a short heating step (10 min at 90–95 °C). The leachable particulate metals accounted for 7–13% of the total particulate phase for Al, 22–37% for Fe, and 68–98% for Mn in coastal waters off Oregon and Washington. Although we have no data allowing for a comparison of Berger’s method and our method, it seems that our method with HCl and long storage time caused a higher dissolution of the aluminosilicate fraction. Thus, our method may not be suitable to evaluate the concentration of bioavailable metals.

The enrichment factor (*EF*) of dissolved metal (dM) in seawater over that of upper crust is defined in a similar manner to that of the metal in aerosols^42^, as follows:

1 $$EF({\rm{dM}})={(\mathrm{dM}/\mathrm{dAl})}_{{\rm{seawater}}}/{(M/\mathrm{Al})}_{{\rm{upper}}{\rm{crust}}}$$

the (M/Al)_upper crust_ is calculated in moles using concentrations published in a previous review^1^. The median is 7.1 (*n* = 436) for *EF*(dFe), which corresponds to 1.3 × 10^2^ for *EF*(dMn), 3.2 × 10^2^ for *EF*(dCo), and 1.2 × 10^3^ for *EF*(dPb)^37^ (Supplementary Fig. 6). The median of *EF*(dPb) was in the same order of magnitude as the *EF* of Pb in aerosols in previous studies^42^. In contrast, *EF*(dMn) and *EF*(dCo) were 10 to 100 times higher than the *EF* of Mn and Co in aerosols^37^. Conversely, the median of *EF*(dFe) was as low as the *EF* of Fe in aerosols (1–2). It is possible that the solubility of trace metals in aerosols affects the *EF*(dM). As an example, the mean solubility was 5.1% for Al, 45% for Pb, 49% for Mn, 36% for Co, and 7.7% for Fe in the East China Sea^43^. These results suggest that the solubility may not be the major factor controlling the trend in the *EF*(dM). Instead, the *EF*(dM) is mainly controlled by the sources of trace metals: indicating that anthropogenic aerosols are a major source for dPb, reductive sources in the ocean, such as sediments on the shelf, are important to Mn and Co, and lithogenic sources are dominant for Fe. *EF*(lpFe) exhibited a median of 2.9, which is also close to the *EF* of Fe in aerosols and re-emphasizes the importance of lithogenic sources. These results are consistent with those of a recent study: total Fe deposition over global oceans from the atmosphere was estimated to be 8.4 Tg y^−1^, most of which is due to mineral dust aerosols except for 7% originating from combustion sources^44^.

The lpFe/tdFe ratio was 0.64 ± 0.23 (average ± sd, *n* = 625). Supplementary Fig. 7a shows a box plot of the lpM/tdM ratio for each metal and indicates that the lpFe/tdFe ratio was as high as the lpAl/tdAl ratio. Supplementary Fig. 7b shows the vertical profile of lpFe/tdFe and demonstrates the general dominance of lpFe in tdFe, with a minimum at intermediate depths due to the remineralization of dFe from settling particles. Major factors that control the lpM/tdM ratio include uptake by phytoplankton and adsorption of dM onto particles. The adsorption may be controlled by surface complexation^45^. Aluminosilicates, manganese oxides, and iron hydroxides have hydroxide groups on the surface to form surface complexes with metal ions. A study on sinking particles in the deep subtropical Atlantic Ocean indicated that the adsorption of metal ions on the particles is controlled by surface complexation with organic coatings^46^. In both cases, the stability of the surface complexes exhibits a linear relationship with the first hydrolysis constant for the metal ion. Because Fe and Al form trivalent cations in seawater, they exhibit the highest hydrolysis constants and lpM/tdM ratios.

**Figure 2:**
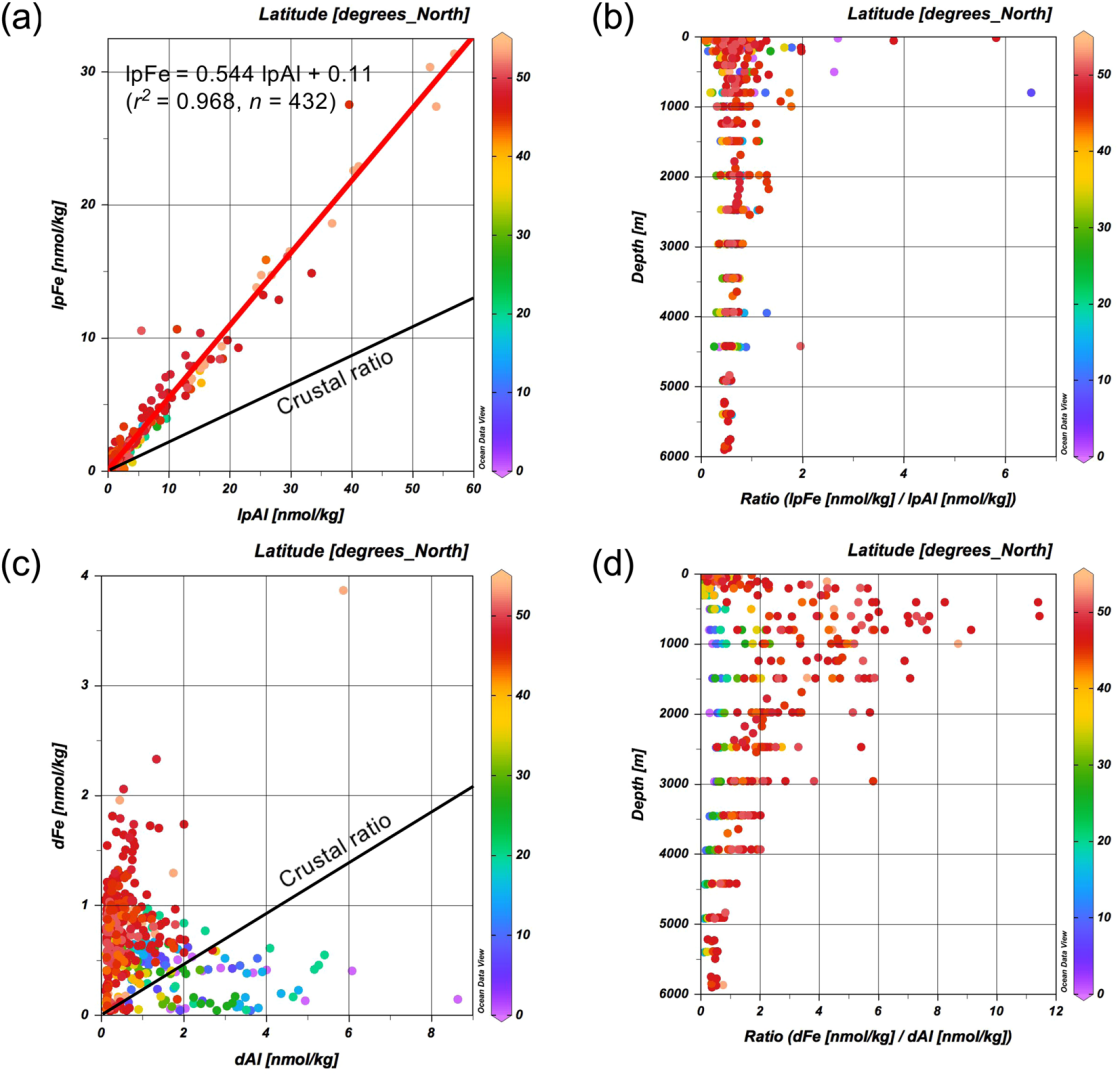
Relationships between lpFe and lpAl, and between dFe and dAl in the North Pacific Ocean. (**a**) Plot of lpFe vs lpAl; (**b**) Vertical distribution of the lpFe/lpAl ratio; (**c**) Plot of dFe vs dAl; (**d**) Vertical distribution of the dFe/dAl ratio. The color of the dots denotes the latitude.

The lpFe exhibited a strong linear relationship with lpAl (Fig. 2a) as follows:

2 $$\begin{array}{c}{\rm{lpFe}}[{\rm{nmol}}\,{{\rm{kg}}}^{-1}]=(0.544\pm 0.005)\,{\rm{lpAl}}\,[{\rm{nmol}}\,{{\rm{kg}}}^{-1}]+0.11\pm 0.04\\ ({r}^{2}=0.968,n=432)\end{array}$$

Significantly high deviations from the regression line were observed near the Aleutian Islands and the Juan de Fuca Ridge^23^, where the lpFe concentration was elevated. A similar linear relationship between Fe and Al was observed in marine aerosols^47^. The slope was 0.25 on a mole basis, meaning that the slope for lp species was approximately twice that for aerosols. This is potentially due to biological uptake and preferential scavenging of Fe in the ocean. The vertical distribution of lpFe/lpAl is shown in Fig. 2b. The lpFe/lpAl ratio was 0.68 ± 0.50 (*n* = 432). This average was of the same order of magnitude as the Fe/Al ratios in crust (0.23), Chinese Loess (0.28), and aerosols from the North Pacific (0.53)^48^. Relatively high ratios above a depth of 1000 m may reflect biogenic particles. High ratios in the depth range of 2000–2500 m reflect scavenged particles from hydrothermal plumes near the Juan de Fuca Ridge^23^. The observed range of lpFe/lpAl ratios in this study appears representative of oceans generally since it encompasses the reported Fe/Al ratios in suspended particles, including 0.30–0.41 from the North Pacific Gyre^18^ and 0.27–0.33 from the Sargasso Sea^49^.

In contrast, dFe and dAl did not show a clear correlation on a basin-scale (Fig. 2c). The data from lower latitudes were generally plotted below the line of the upper continental ratio while those from higher latitudes were plotted above the line. The dFe/dAl ratio was 0.30 ± 0.23 (*n* = 68) below 20°N, 2.1 ± 2.1 (*n* = 339) above 20°N, and 1.8 ± 2.0 (*n* = 407) for all data (Fig. 2d). Thus, the high dFe/dAl ratio is a characteristic of the northern North Pacific Ocean. Specifically, the high dFe/dAl ratio occurred at intermediate depths. The dFe/dAl ratio was less than 0.9 below a depth of 4500 m where LCDW flows. Supplementary Fig. 8a,b shows the horizontal distribution of dFe and phosphate, respectively, at a depth of 4500 m. It is evident that the horizontal distribution of dFe was distinct from that of phosphate. The concentrations of dFe and phosphate below a depth of 4500 m are plotted against latitude in Supplementary Fig. 8c,d, respectively. The concentration of phosphate increased from south to north and reflects the biogeochemical accumulation of phosphate in older deep water. Although dFe increased above 20°N in a similar manner to phosphate, it exhibited a relatively constant concentration below 20°N. The increase in dFe above 20°N may reflect increasing regenerated nutrients or margin sources. The concentration of dAl generally decreased from south to north and reflects the supply by LCDW and scavenging that occurred en route^37^. In addition, dAl exhibited a maximum near the Hawaiian Island Chain as well as dFe. The dFe/dAl ratio below a depth of 4500 m was about 0.11 below 20°N (Supplementary Fig. 8e). Thus, it is likely that the lithogenic source of dFe as well as more particulate reactive nature of dFe is a cause of decoupling between dFe and phosphate in their distributions in North Pacific deep water.

**Figure 3:**
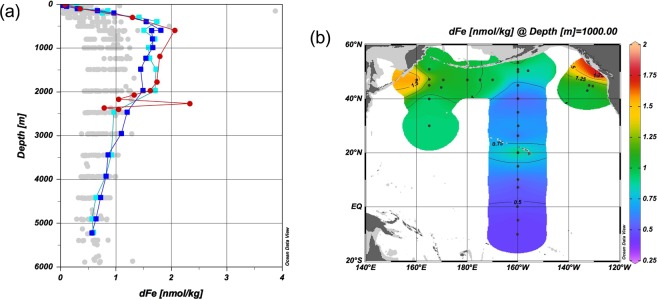
Distribution of dFe in the North Pacific Ocean. (**a**) Vertical profiles of dFe. Blue squares and cyan squares represent TR16 and BD07, respectively, at 160°E, 47°N; red circles represent BD21 at 128.713°W, 48.454°N; gray circles represent the other stations. (**b**) Horizontal distribution of dFe at a depth of 1000 m.

A significant feature in the sectional distributions of dFe along 165°E and 47°N was the maximum observed in the depth range of 400–2000 m at the westernmost stations of TR16 and BD07 (Fig. 1). Nishioka *et al*.^20,50^ explained that the intermediate-depth Fe maximum is caused by the supply of Fe from the Sea of Okhotsk and by the intensive tidal mixing in the Kuril Straits in the context of formation of the Okhotsk Sea Intermediate Water. However, in the present study, a similar intermediate-depth maximum of dFe occurred at the easternmost station BD21, located approximately 200 km off Vancouver Island (Fig. 3a), suggesting that this is a more general feature of dFe. Additionally, the corresponding intermediate-depth maxima for phosphate, dAl, and lpFe were unclear (Supplementary Figs 9 and 10). Continental shelf slope sediments are well known as sources of dFe^21,51–55^. We propose that a combination of effects around the continental slope produced the observed unique distribution of dFe. Manganese reduction in continental shelf sediments is the most important source of dMn to the North Pacific, and dMn spreads mainly via NPIW^37^. When manganese oxide is reduced, dFe is desorbed from the oxides and transferred along with dMn. The concurrent appearance of dMn and dFe was observed on the Bering Sea shelf ^56^, and dFe and dMn exhibited a similar horizontal distribution at a depth of 1000 m in the North Pacific (Fig. 3b and Supplementary Fig. 9f). In the depth range of 500–1800 m, dFe exhibited the highest linear correlation with dMn (*r* = 0.812 ± 0.003, *n* = 134; Supplementary Fig. 11). This process can supply a large amount of dFe to the continental margins, leading to the luxury uptake of Fe by phytoplankton^57^. In Supplementary Fig. 12a,b, dFe and phosphate are plotted against the apparent oxygen utilization (AOU) for depths less than 500 m. The slopes of the linear regressions of dFe–AOU were relatively high (0.00367–0.00487) at stations TR16, BD07, and BD21, suggesting remineralization of dFe from Fe-rich organic particles. Therefore, it can be concluded that dFe was selectively enhanced in a depth range of 400–2000 m at stations TR16, BD07, and BD21, forming pronounced intermediate depth maxima of the dFe/phosphate ratio (Supplementary Fig. 12c,d).

**Figure 4:**
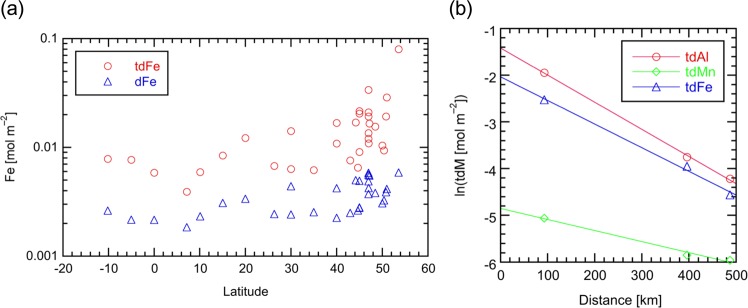
Distribution of the integral concentrations of tdFe and dFe in the North Pacific Ocean. (**a**) Latitudinal distribution of the integral concentrations of tdFe and dFe at all stations in this study. (**b**) The natural logarithm of the integral concentrations of tdAl, tdMn, and tdFe plotted relative to distance along 160°W from the shelf break of the Aleutian Islands. The lines denote linear regressions.

In order to evaluate the geochemistry of tdM and dM on the basis of the standing stock, we calculated the integrated concentrations throughout the water column at each station. The integrated concentrations of tdFe and dFe are plotted relative to latitude in Fig. 4a. The integrated concentrations of tdFe were 2.1–13.6 times larger than those of dFe. Below 45°N, our stations were located in the interior of the ocean where variations in the integrated concentrations were relatively low. Integrated concentrations significantly increased at continental margins and similar trends were observed for Al, Mn, and Co (Supplementary Fig. 13). Supplementary Fig. 14a,b shows the dependency of the integrated concentrations of Al, Mn, and Fe in d and td fractions, respectively, relative to distance along 160°W from the shelf break of the Aleutian Islands. The integrated concentrations of tdM and dM sharply decreased within 500 km. The natural logarithms of the integrated concentrations of tdM and dM decreased linearly with respect to distance (Fig. 4b and Supplementary Fig. 14c), suggesting the removal of tdM and dM. Thus, we propose that the boundary scavenging zone has a width of 500 km from the Aleutian shelf. This is the first attempt of using the integrated concentrations of Al, Mn, and Fe to identify the boundary scavenging zone, although the decrease in the concentration of dFe over continental shelves has been previously discussed^58^. The boundary scavenging zone has been evaluated from data on scavenged elements, including ^210^Pb and ^231^Pa^59,60^.

We assumed that a width of 500 km is typical for the boundary scavenging zone, and estimate that the boundary scavenging zone and ocean’s interior occupy 16% and 84%, respectively, of the total area of the North Pacific corresponding to 7.70 × 10^7^ km^2 ^^16^. We calculate the average integral concentrations for the boundary scavenging zone and interior and obtain the inventory for tdM and dM (Supplementary Table 4). The total inventory in the North Pacific is equal to 1.1 × 10^12^ mol for tdFe and 2.8 × 10^11^ mol for dFe. The difference of 8.2 × 10^11^ mol equates to the amount of lpFe. These are the first estimates of inventories of Fe fractions in the North Pacific on the basis of observations. The large quantity of lpFe further emphasizes the potential importance of lpFe in the ocean iron cycle. A large portion of lpFe may not be bioavailable, as discussed above. However, given that protozoan grazers remineralize particulate Fe^61–63^, the importance of lpFe may have been underestimated as a source of bioavailable Fe. The quantitative estimation of this process, taking account of a time-scale and a flux, will be a challenge to future work.

## Methods

### Sampling and analysis

Detailed descriptions of the methods and hydrography are reported in a previous study^37^. Seawater samples were collected during the three GEOTRACES Japan cruises (KH-05-2, KH-11-7, and KH-12-4) by using a clean sampling system equipped with Niskin-X bottles (General Oceanics, USA). During KH-05-2 and KH-11-7, a portion of seawater sampled for the dissolved species was passed through a 0.2 µm Nuclepore filter (Whatman, UK) using a closed filtration system, and then acidified with ultrapure HCl (Tamapure AA-10, Tama Chemicals, Japan) to achieve a final concentration of 0.010 mol kg^−1^ HCl with pH 2.2. The portion of seawater sampled for the total dissolvable species analysis was acidified without filtration. During KH-12-4, filtration was conducted by using an AcroPak capsule filter with a pore size of 0.2 µm (Pall, USA), which was directly attached to a Niskin bottle. The seawater samples were immediately acidified in the manner described above, and stored at ambient temperature for at least 1 y prior to analysis. We did not apply UV irradiation to the samples to avoid contamination of the trace metals and a possible undesired interference on their analysis.

An off-line automated solid-phase extraction system (SPE-100, Hiranuma Sangyo, Japan) equipped with a column of Nobias Chelate-PA1 resin (Hitachi High Technologies, Japan) was used for the preconcentration of trace metals from seawater^36^. The trace metals were eluted with 1.0 mol kg^−1^ HNO_3_ (Optima Acids, Thermo Fisher Scientific, USA) and were determined with a high-resolution inductively coupled plasma mass spectrometer (HR-ICP-MS, Element 2, Thermo Fisher Scientific) via the calibration curve method.

We evaluated the procedure blanks by using deionized water prepared with a Milli-Q system (Merck Millipore, Germany) as a sample. We defined the detection limits for total dissolvable metal (tdM) and dissolved metal (dM) as three times the standard deviation (sd) of the procedure blank. Given that the relative standard deviation (rsd) was approximately 5% for both tdM and dM, the detection limit of labile particulate metal (lpM) was defined by using the following equation by considering the propagation of uncertainty: 2 × √2 × 0.05 × *C*_ave_, where *C*_ave_ denotes the average concentration of dM in the study. The procedure blanks and detection limits for Fe are summarized in Supplementary Table 5. To evaluate the accuracy of our measurements, we analyzed the certified reference materials for trace metals (i.e., CASS-5 and NASS-6), as well as the GEOTRACES open-ocean reference samples GS and GD at an early point of the study^36^. We participated in the intercalibration campaign of new reference materials of CASS-6 and NASS-7, and contributed to the establishment of certified values at a later point of the study^64^. We used the Ocean Data View software^65^ for analyzing data and plotting figures.

### Inter- and intra-comparison of vertical profiles

We compared our Fe data from ST09 (30.007°N, 159.996°W) with data from the Vertex IV site (28°N, 155°W)^18^ in Supplementary Fig. 5. The authors collected particulate samples on a 0.3 µm Nuclepore filter, and analyzed them for trace metal content of the 25% acetic acid leachate and residual refractory fraction treated with HCl–HNO_3_–HF. The dFe and lpFe at ST09 slightly exceeded the dFe and total particulate Fe (pFe) at Vertex IV, respectively. Similar results were observed for Al and Mn^37^. It is possible that the Hawaiian Island Chain is a source of the elevated Fe, Al, and Mn at ST09 because the Hawaiian Island Chain is located approximately 800 km southwest of ST09.

In the GEOTRACES program, a crossover station is a location where the track of one cruise overlaps with that of another cruise, although the research vessels need not be simultaneously present at the same location (http://www.geotraces.org). A comparison of the results from crossover stations provides a measure of internal consistency. Strict crossover stations were absent between cruises KH-05-2 and KH-12-4. However, stations ST13 and 14 of KH-05-2 were located approximately 100 km and 300 km from BD15 of KH-12-4 (160°W, 50.8°N), respectively. Thus, we used the vertical profiles at these stations to attempt an intracomparison. The upper panels in Supplementary Fig. 15 show the vertical profiles of tdFe and dFe at these stations. Higher concentrations of tdFe and dFe were observed at ST14, which was located approximately 80 km off the shelf break and above the Aleutian Trench. The high concentrations of Fe in the surface water were supplied by the Alaskan Stream from the continental shelf. In deep water, Fe was supplied by the settling and remineralization of biogenic particles and resuspension of sediments from the continental slope. Specifically, the integral concentration of tdFe and dFe decreased logarithmically with respect to distance from the shelf, thereby indicating boundary scavenging.

With respect to KH-11-7 and KH-12-4, stations TR16 and BD07 were at the same position (160°E, 47°N) as a crossover station. With respect to dFe, the vertical profiles were generally consistent (Supplementary Fig. 15), and a strong linear relationship existed between TR16 and BD07 (Supplementary Fig. 16). However, tdFe in 2011 exceeded that in 2012. The same trend was observed for Al and an enhanced bottom concentration of Mn was observed in 2012^37^. The changes of Fe and Al could be due to variations of the water mass movements in the area from year-to-year, or to the presence of eddies that carry a different concentration into the region. However, we propose that the observed changes were related to the earthquake off the Pacific coast of Tohoku, Japan (with a magnitude of 9.1) that occurred at 38.297°N, 142.373°E on March 11, 2011 and was followed by a tsunami and turbidity currents^37^. Given that the distance between the seismic center and TR16 was approximately 1,730 km, the disturbance on Fe and Al concentrations would have reached TR16 prior to sampling within five months. In addition, the high Mn concentrations in 2012 can be explained by development of a temporal reductive source on the bottom sediments where a large amount of organic detritus had been transported by the tsunami and turbidity currents, followed by release and transport of dMn.

The samples from these crossover stations were stored for different time periods (1–10 y) until the preconcentration was performed. However, the effect of storage time was undetectable. This result is one of the reasons for us to conclude that both the unfiltered and filtered seawater samples at pH 2.2 were stable for the determination of tdM and dM from 1 to 10 y.

## Supplementary information

Supplementary Table 1

Supplementary Information
