# Supplementary material for: Major lithogenic contributions to the distribution and budget of iron in the North Pacific Ocean
Source: Sci Rep. 2019 Aug 12;9:11652. doi: 10.1038/s41598-019-48035-1 (PMC6690902; doi:10.1038/s41598-019-48035-1)
Supplement: Supplementary file 2 — Supplementary Information [file 41598_2019_48035_MOESM2_ESM.pdf]

Supplementary Information

Major lithogenic contributions to the distribution and budget of iron in the North Pacific Ocean

Linjie Zheng [1] and Yoshiki Sohrin [1]*

[1] Institute for Chemical Research, Kyoto University, Gokasho, Uji, Kyoto 611-0011, Japan

*Corresponding author: Y. Sohrin. E-mail address: sohrin@scl.kyoto-u.ac.jp

Supplementary Figure Captions

Fig. 1. Map of the study area with location of the sampling points.

Fig. 2. Sectional distribution of dFe and lpFe in the depth range of 0–1000 m along 160°W, 165°E, and 47°N. EUC: Equatorial Under Current; NEC: North Equatorial Current; AS: Alaskan Stream; STMW: Subtropical Mode Water; CMW: Central Mode Water; NPIW: North Pacific Intermediate Water; EqPIW: Equatorial Pacific Intermediate Water; OSMW: Okhotsk Sea Mode Water.

Fig. 3. Sectional distribution of tdFe along 160°W, 165°E, and 47°N. The potential density isolines of 26.4 and 27.2 represent the upper and lower boundaries of the North Pacific Intermediate Water (NPIW), and those of 27.8 represent the upper boundary of the Lower Circumpolar Deep Water (LCDW). EqPIW: Equatorial Pacific Intermediate Water; UCDW: Upper Circumpolar Deep Water; PDW: Pacific Deep Water; AS: Alaskan Stream; CMW: Central Mode Water; OSMW: Okhotsk Sea Mode Water.

Fig. 4. Horizontal distribution of lpFe at a depth of 4500 m. The red line shows the major flows of the LCDW, which were obtained from the literature [1]. A circle with a centre point shows upwelling from this layer.

Fig. 5. Vertical profiles of dFe and pFe. Red triangles denote dFe and lpFe at ST09 (30.007°N, 159.996°W). a. Open circles denote dFe at the Vertex IV site (28°N, 155°W) [2]. b. Open circles, squares, and diamonds denote acetic acid leachable, refractory, and total pFe, respectively, at the Vertex IV site.

Fig. 6. a. Box plot of *EF*(dM) for Mn, Fe, Co, and Pb. b. Box plot of *EF*(lpM) for Mn, Fe, Co, and Pb. The middle line of the box denotes the median; the top and bottom lines of the box denote the upper and lower quartiles, respectively; and circles denote potential outliers. Labile particulate Pb was undetected in most of samples [3].

Fig. 7. a. Box plot of the lpM/tdM ratio for each element. The middle line of the box denotes the median; the top and bottom lines of the box denote the upper and lower quartiles, respectively; and circles denote potential outliers. b. Vertical distribution of lpFe/tdFe. The colour of the dots denotes the latitude.

Fig. 8. Horizontal distributions of dFe (a) and phosphate (b) at a depth of 4500 m. Latitudinal distributions of dFe (c), phosphate (d), and the dFe/dAl ratio (e) in deep water below a depth of 4500 m.

Fig. 9. Vertical distributions of phosphate (a), dAl (c), and dMn (e) in the North Pacific. Blue squares and cyan squares represent TR16 and BD07, respectively, at 160°E, 47°N; red circles represent BD21 at 128.713°W, 48.454°N; gray circles represent the other stations. Horizontal distributions of phosphate (b), dAl (d), and dMn (f) at a depth of 1000 m.

Fig. 10. a. Vertical profiles of lpFe. Blue squares and cyan squares represent TR16 and BD07, respectively, at 160°E, 47°N; red circles represent BD21 at 128.713°W, 48.454°N; gray circles represent the other stations. b. Horizontal distribution of lpFe at a depth of 1000 m.

Fig. 11. Plot of dFe vs dMn in the depth range of 500–1800 m in the North Pacific Ocean. Blue squares and cyan squares represent TR16 and BD07, respectively, at 160°E, 47°N; red circles represent BD21 at 128.713°W, 48.454°N; gray circles represent the other stations. The Pearson correlation coefficient for all data is $r = 0.812 \pm 0.003$, $n = 134$.

Fig. 12. Plots of dFe vs AOU (a), and phosphate vs AOU (b), in the depth range of 0–500 m in the North Pacific Ocean. Plot of dFe vs phosphate (c) for all data. Vertical profiles of the dFe/phosphate ratio. Blue squares and cyan squares represent TR16 and BD07, respectively, at 160°E, 47°N; red circles represent BD21 at 128.713°W, 48.454°N; gray circles represent the other stations. The regression lines for dFe vs AOU are as follows: for TR16, dFe = 0.00416

AOU + 0.20, r^2 = 0.958, n = 8; for BD07, dFe = 0.00367 AOU + 0.25, r^2 = 0.828, n = 7; for BD21, dFe = 0.00487 AOU + 0.18, r^2 = 0.960, n = 3.

Fig. 13. Latitudinal distribution of the integral concentrations at all stations in this study for tdAl and dAl (a), tdMn and dMn (b), and tdCo and dCo (c).

Fig. 14. Integral concentrations of tdM (a) and dM (b) plotted against distance along 160°W from the shelf break of the Aleutian Islands. The shaded area denotes the boundary scavenging zone proposed in this study. c. Plots of ln(dM) vs distance. The lines represent linear regressions.

Fig. 15. a. Vertical profiles of tdFe and dFe at ST13 (red circles), BD15 (blue squares), and ST14 (cyan diamonds). b. Vertical profiles of tdFe and dFe at TR16 (red circles) and BD07 (blue squares). The map shows the locations of the stations and the distribution of earthquakes around the Kuril–Kamchatka Trench from February 28 to July 28, 2011. The diameter of the black circles denotes the relative magnitude of the earthquake. The data were obtained from https://earthquake.usgs.gov.

Fig. 16. Linear regression between BD15 and ST13 for tdFe (a) and dFe (b), and between TR16 and BD07 for tdFe (c) and dFe (d).

References

1 Kawabe, M. & Fujio, S. Pacific Ocean circulation based on observation. *J. Oceanogr.* **66**, 389-403, doi: 10.1007/s10872-010-0034-8 (2010).

2 Bruland, K. W., Orians, K. J. & Cowen, J. P. Reactive trace metals in the stratified central North Pacific. *Geochim. Cosmochim. Acta* **58**, 3171-3182, doi: 10.1016/0016-7037(94)90044-2 (1994).

3 Zheng, L. *et al.* Distinct basin-scale-distributions of aluminum, manganese, cobalt, and lead in the North Pacific Ocean. *Geochim. Cosmochim. Acta* **254**, 102-121, doi: 10.1016/j.gca.2019.03.038 (2019).

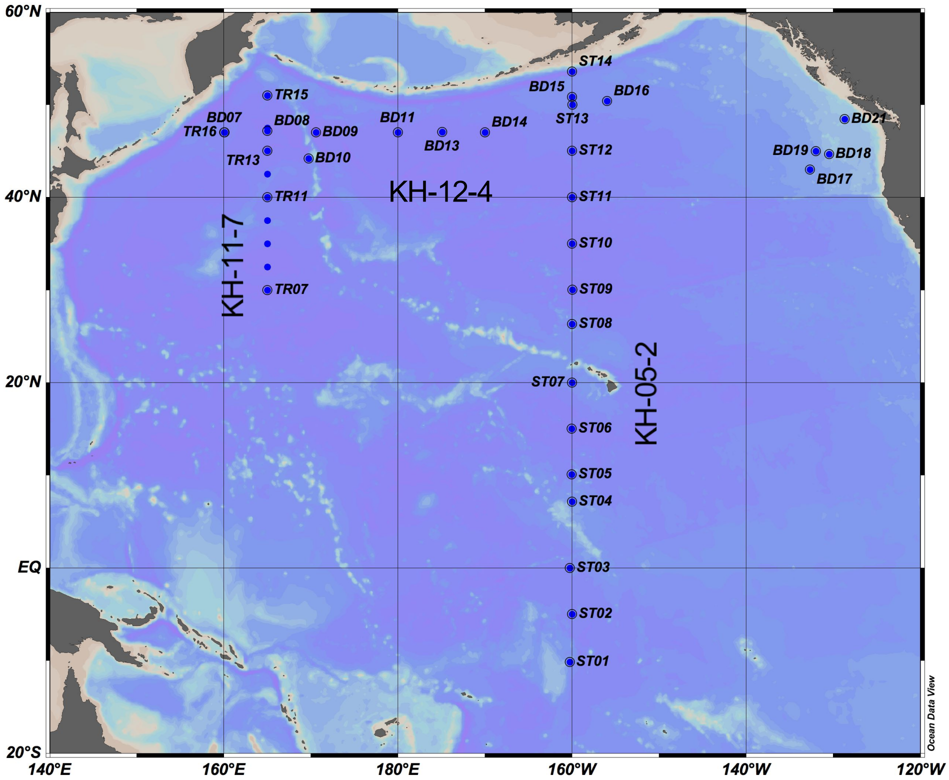

Supplementary Figure 1

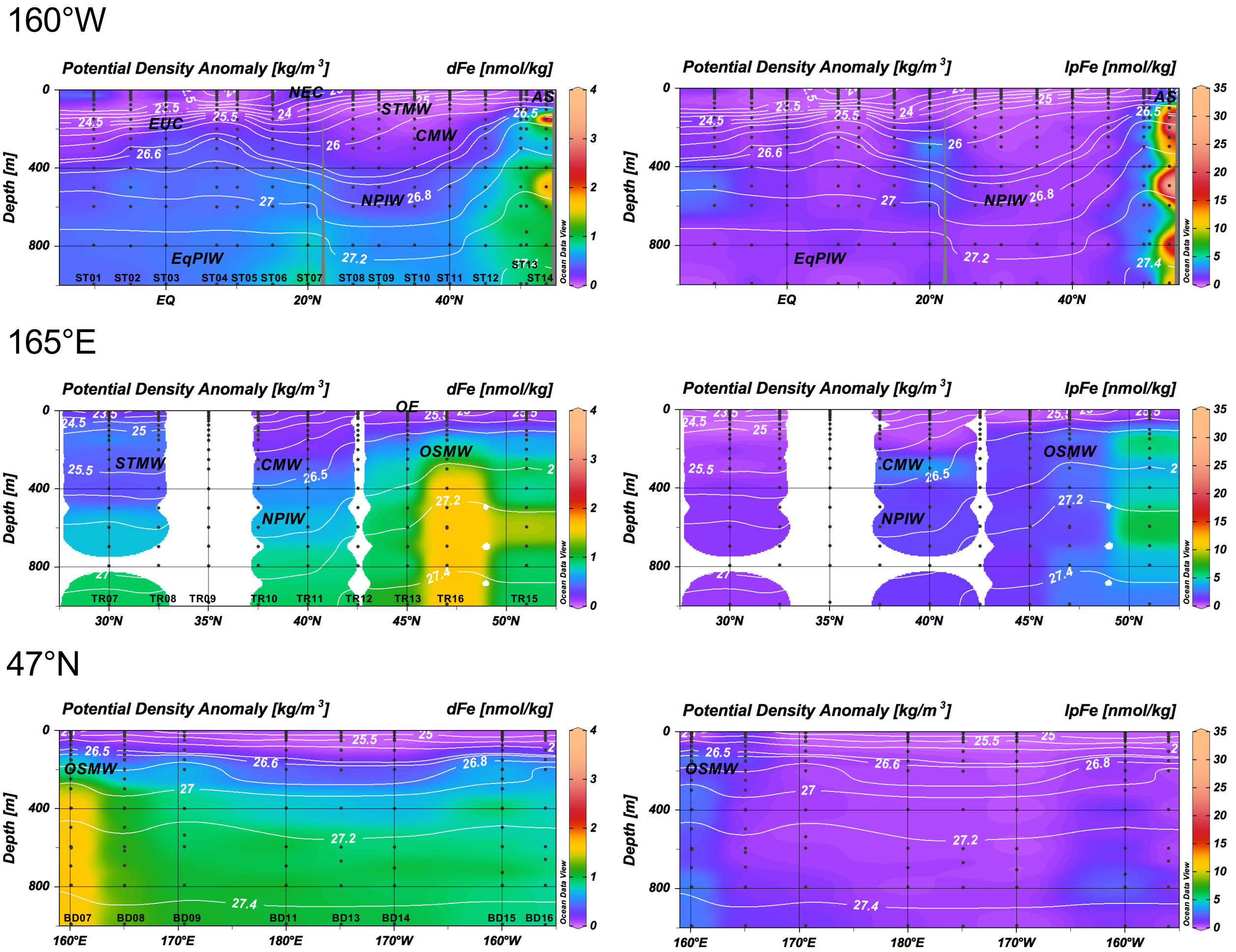

Supplementary Figure 2

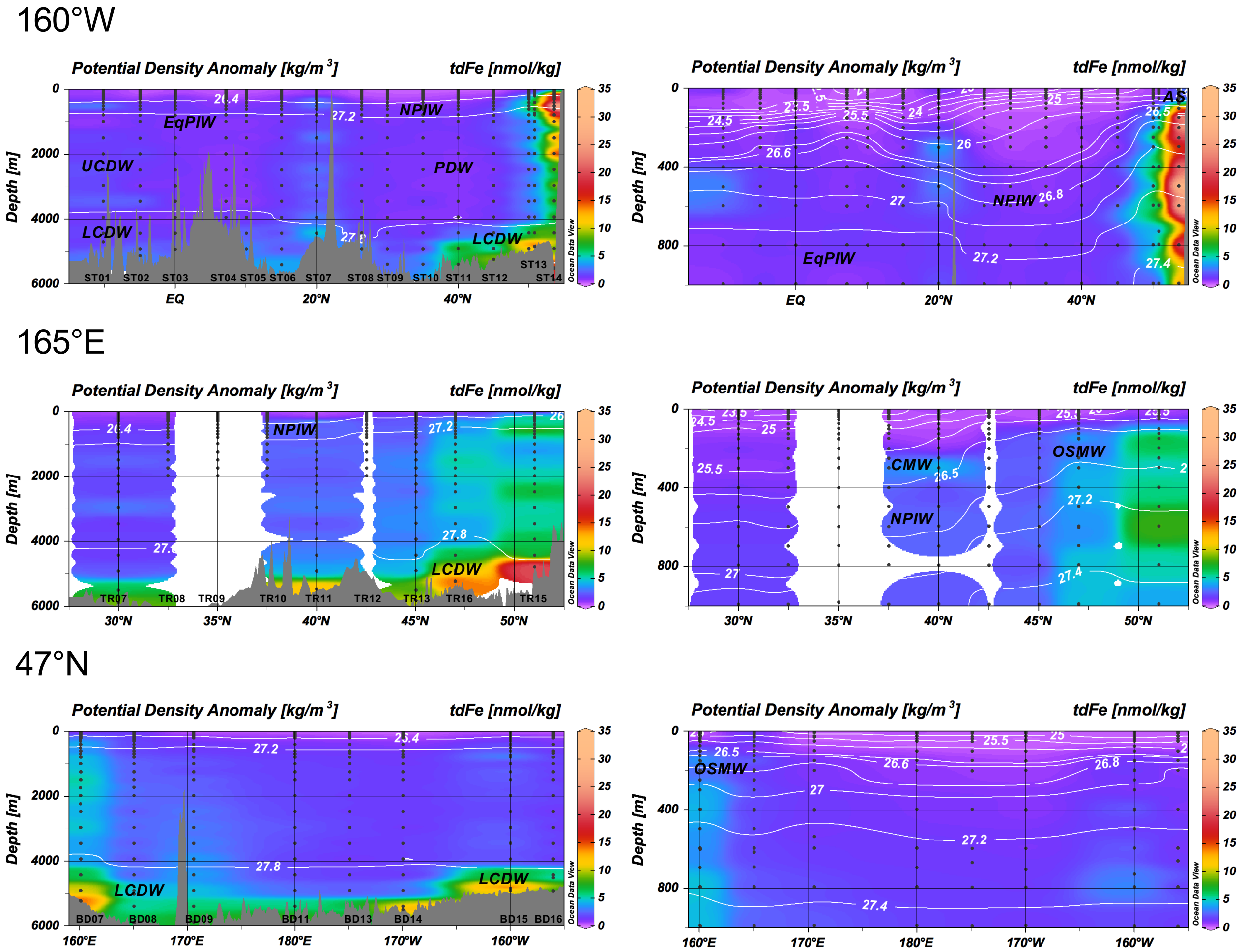

Supplementary Figure 3

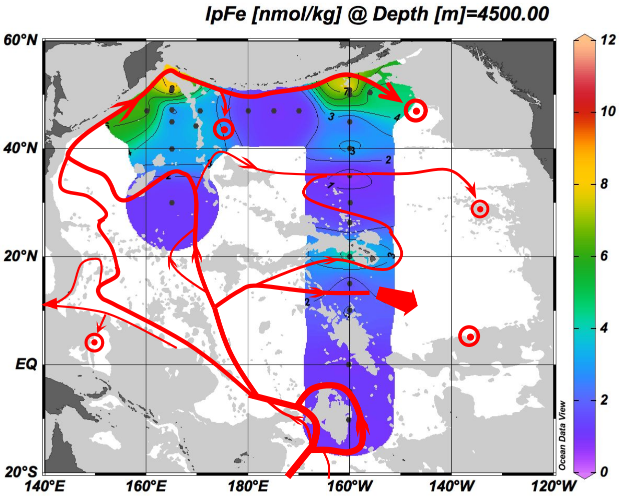

Supplementary Figure 4

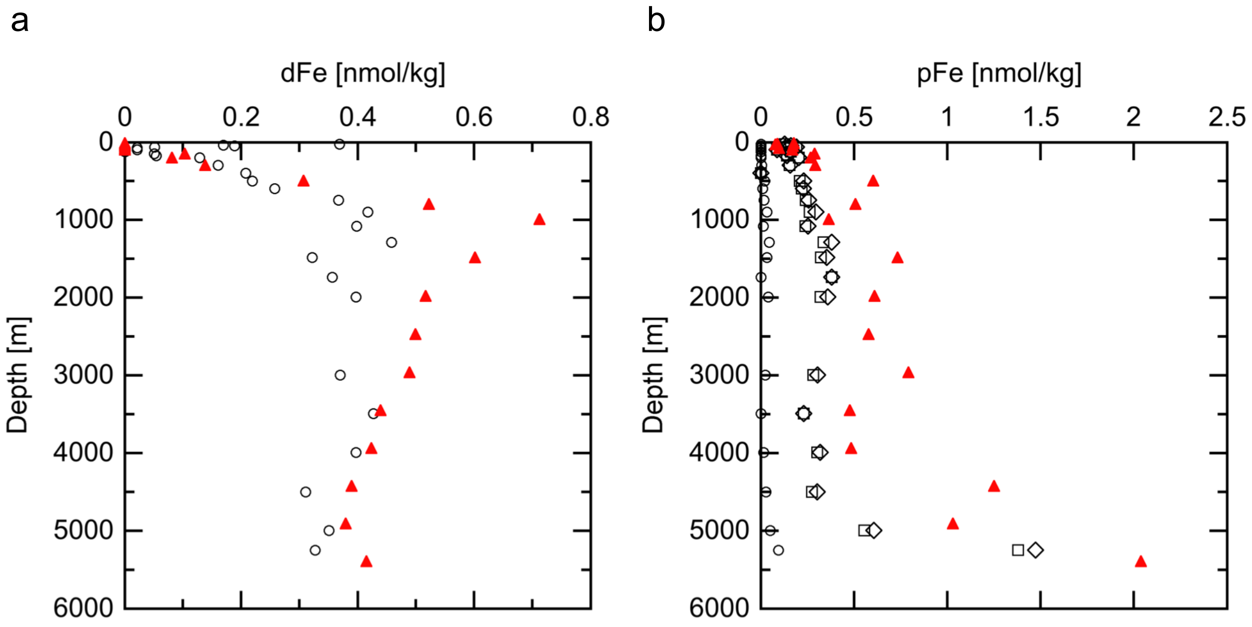

Supplementary Figure 5

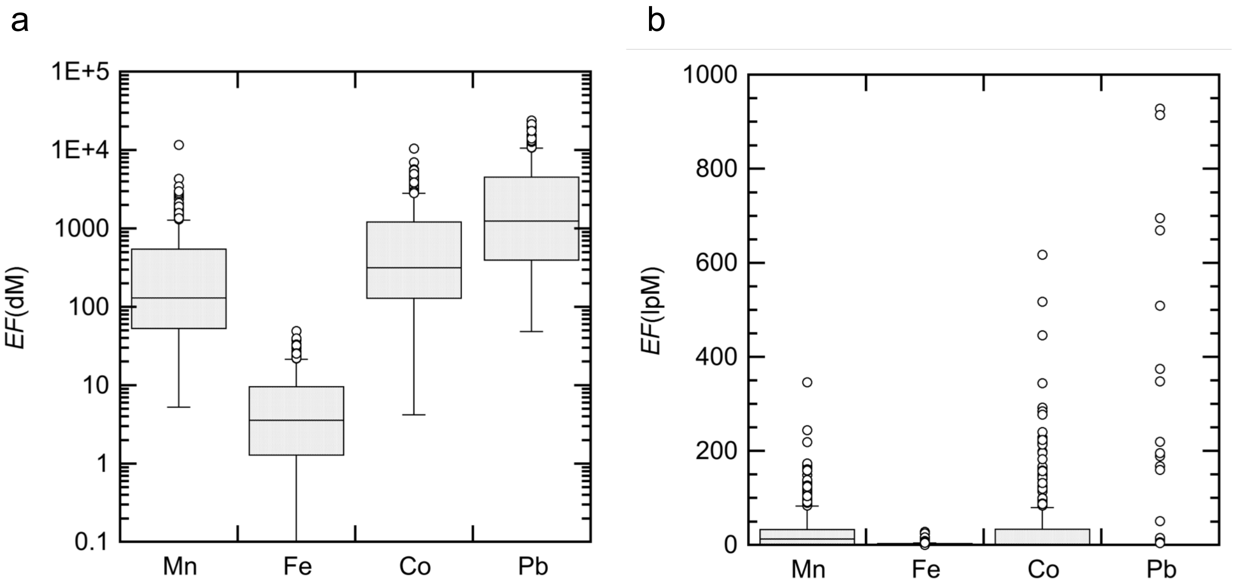

Supplementary Figure 6

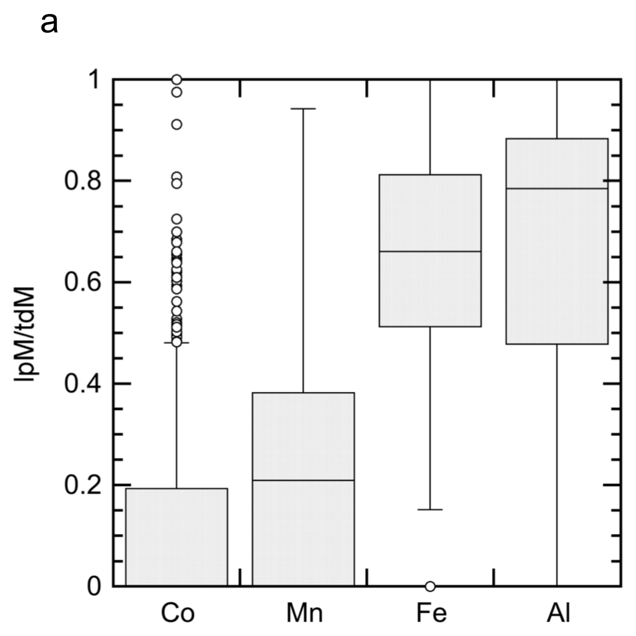

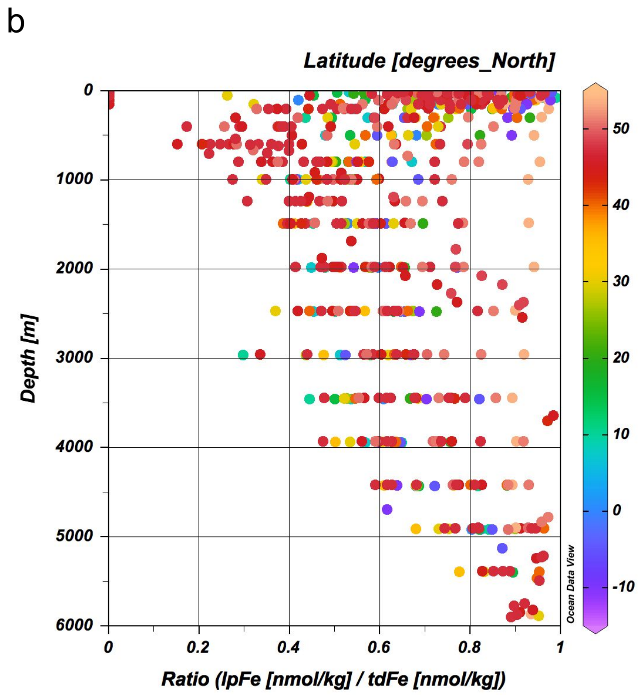

Supplementary Figure 7

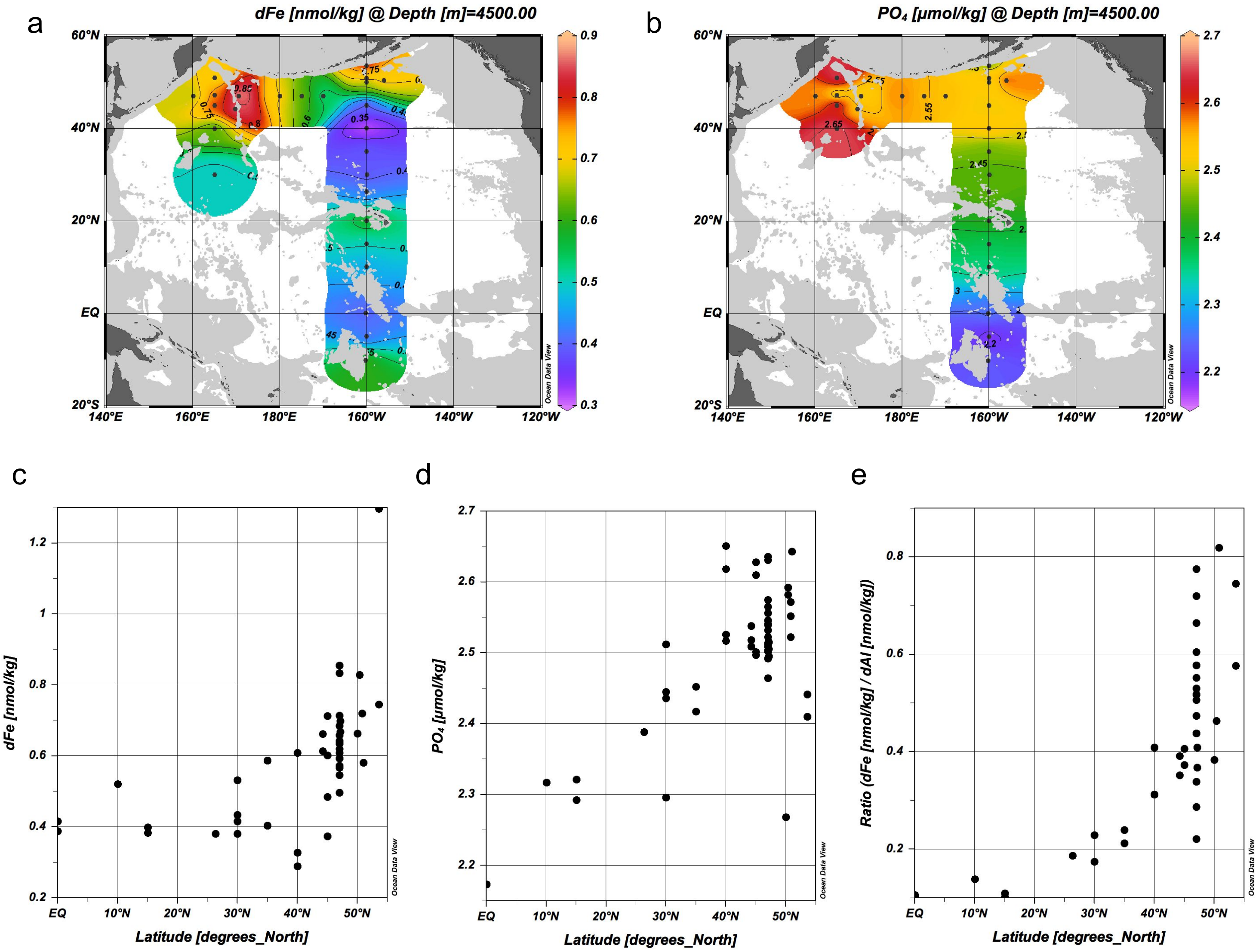

Supplementary Figure 8

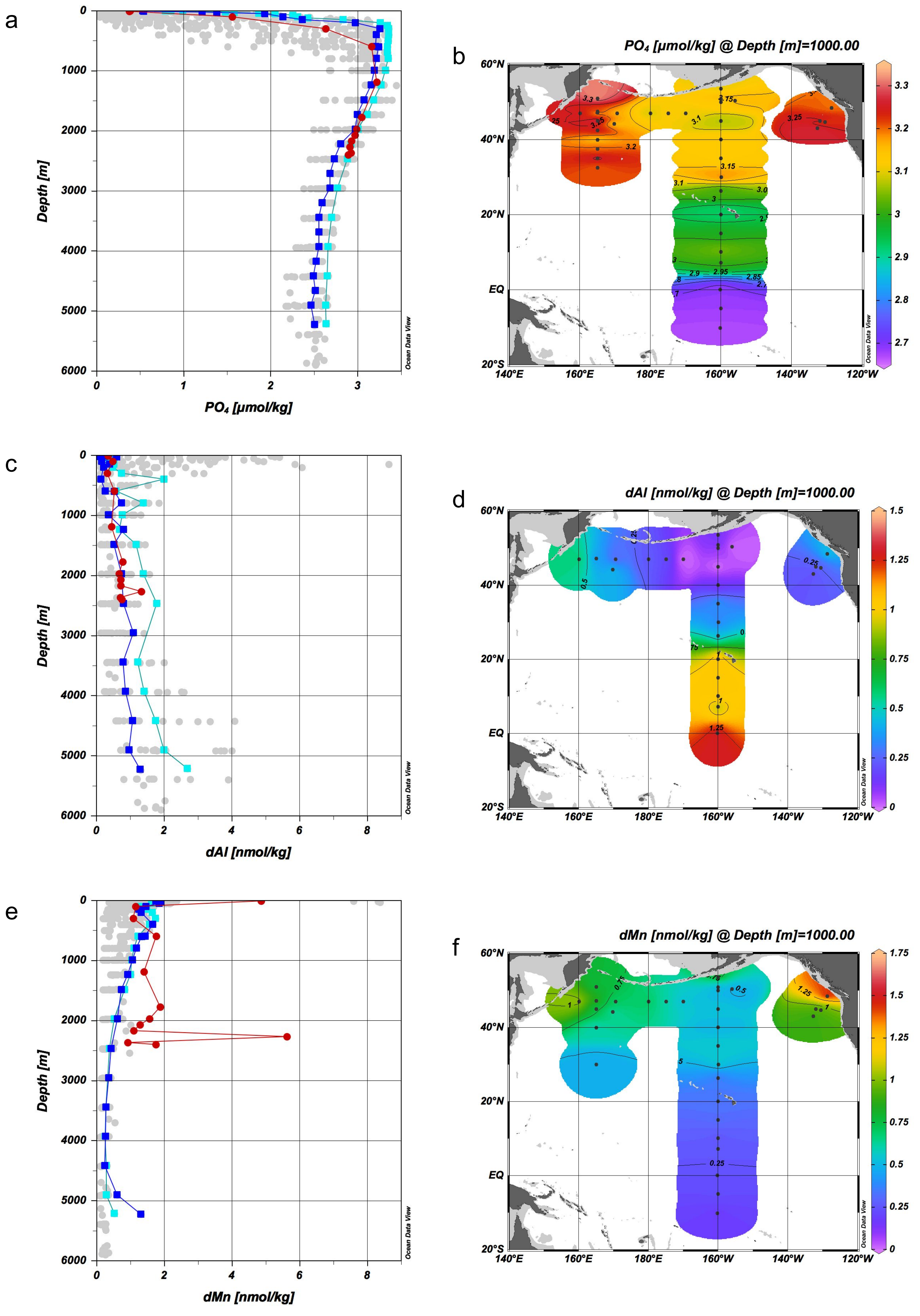

Supplementary Figure 9

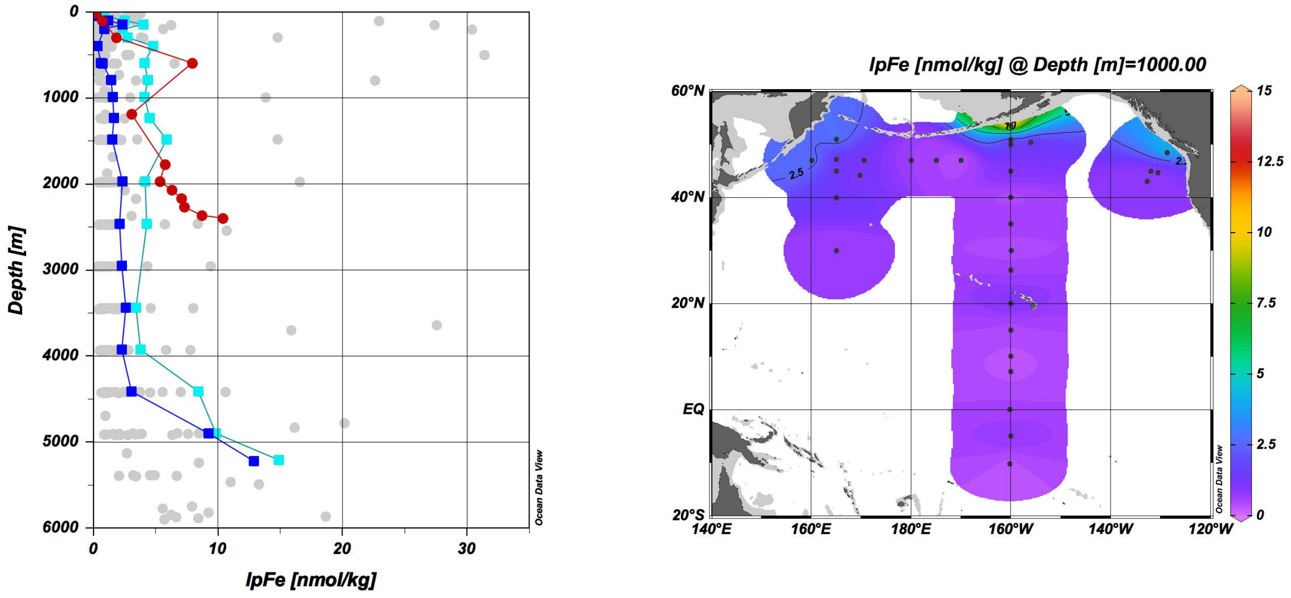

Supplementary Figure 10

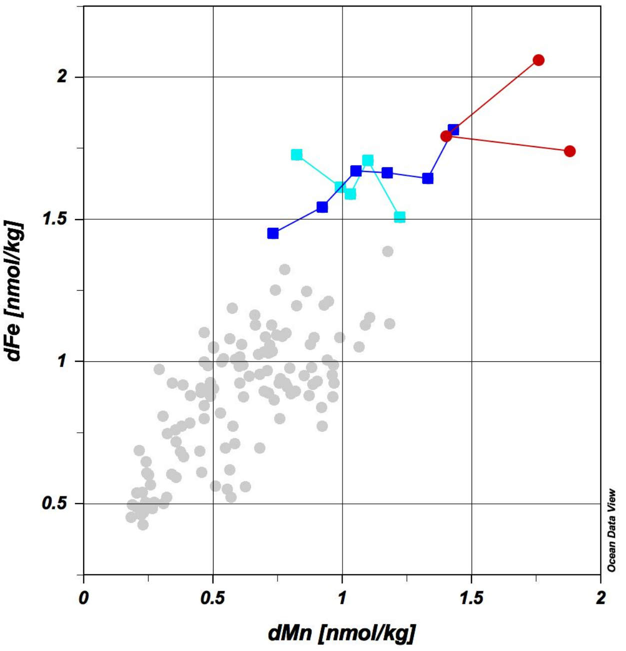

Supplementary Figure 11

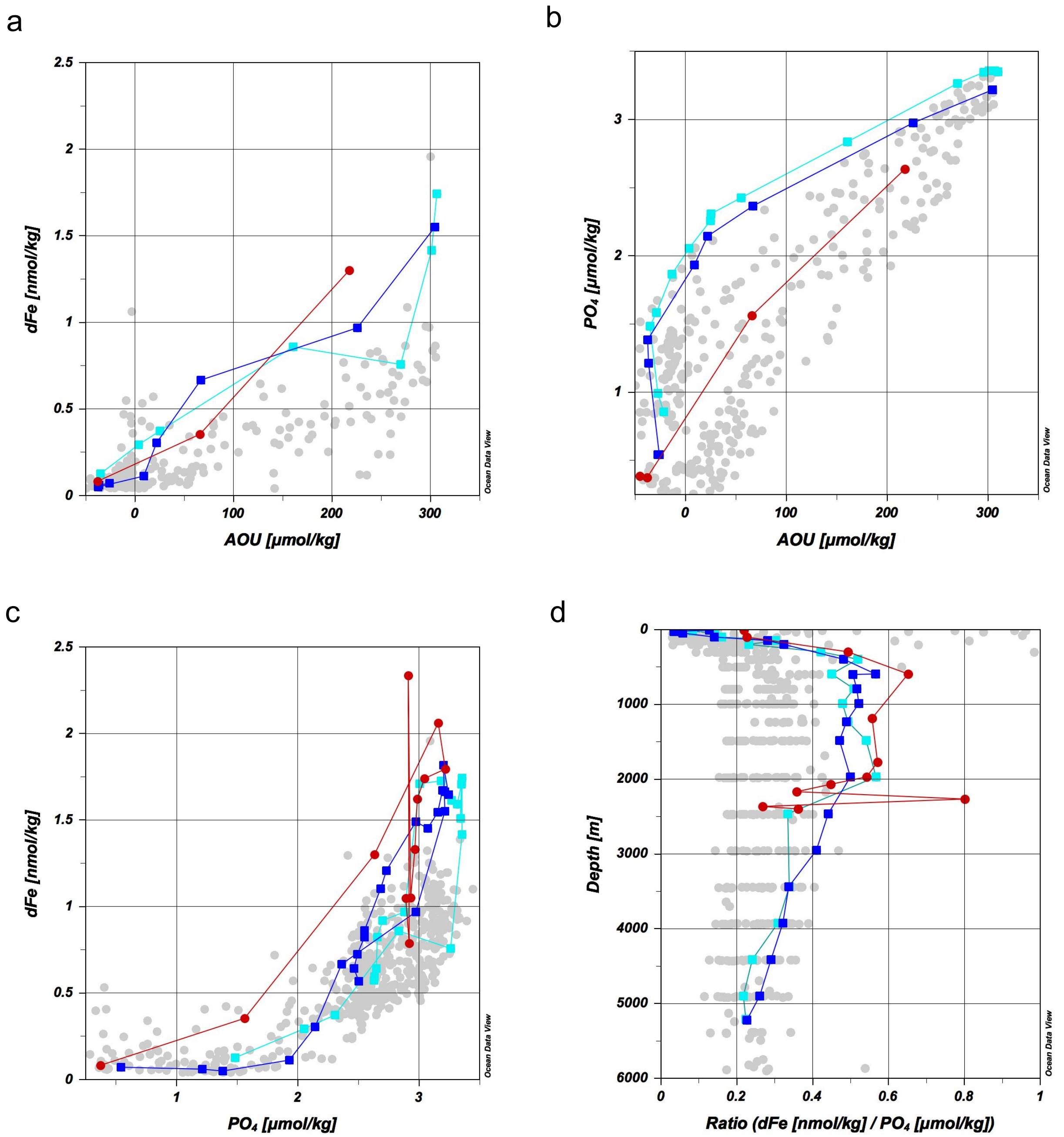

Supplementary Figure 12

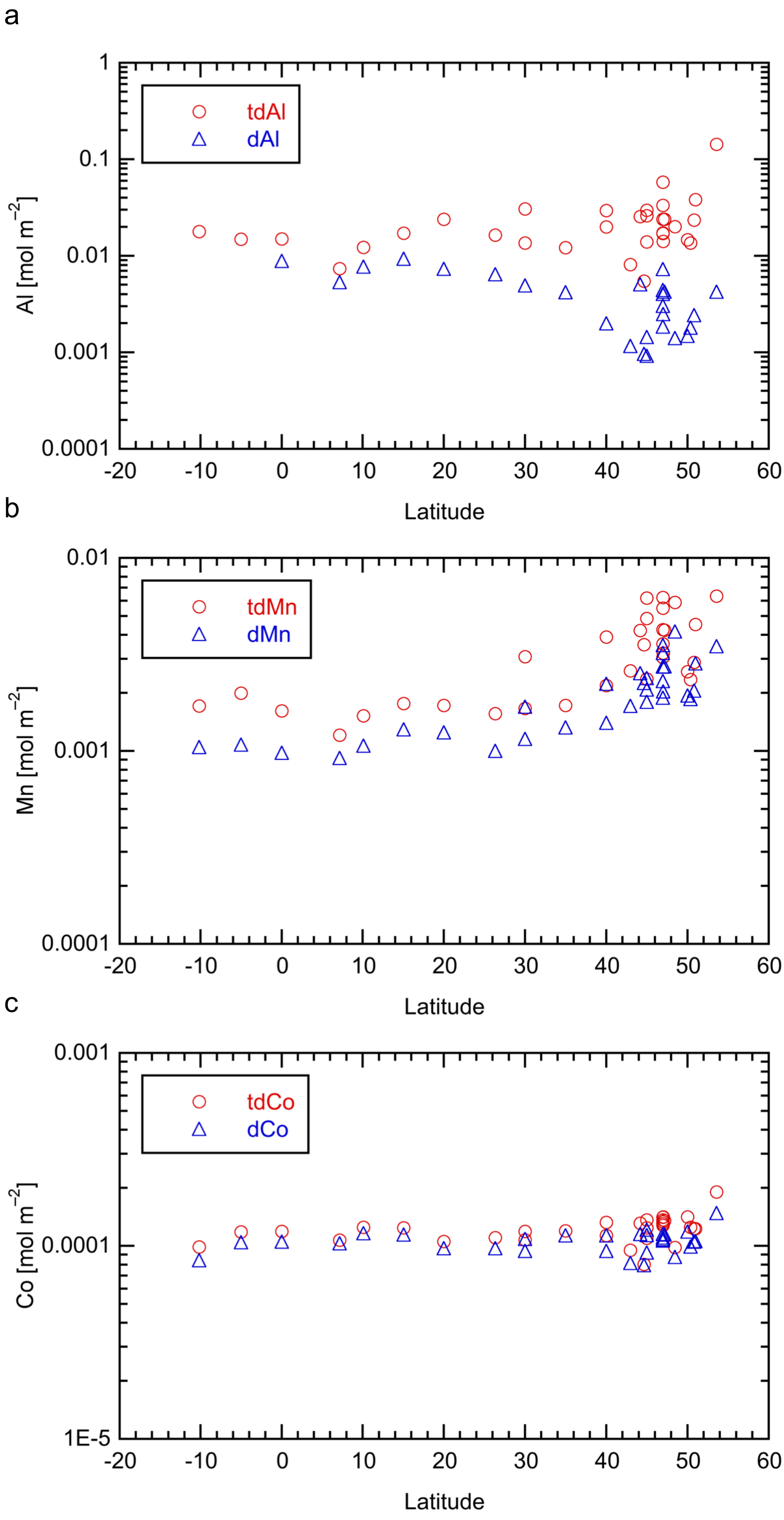

Supplementary Figure 13

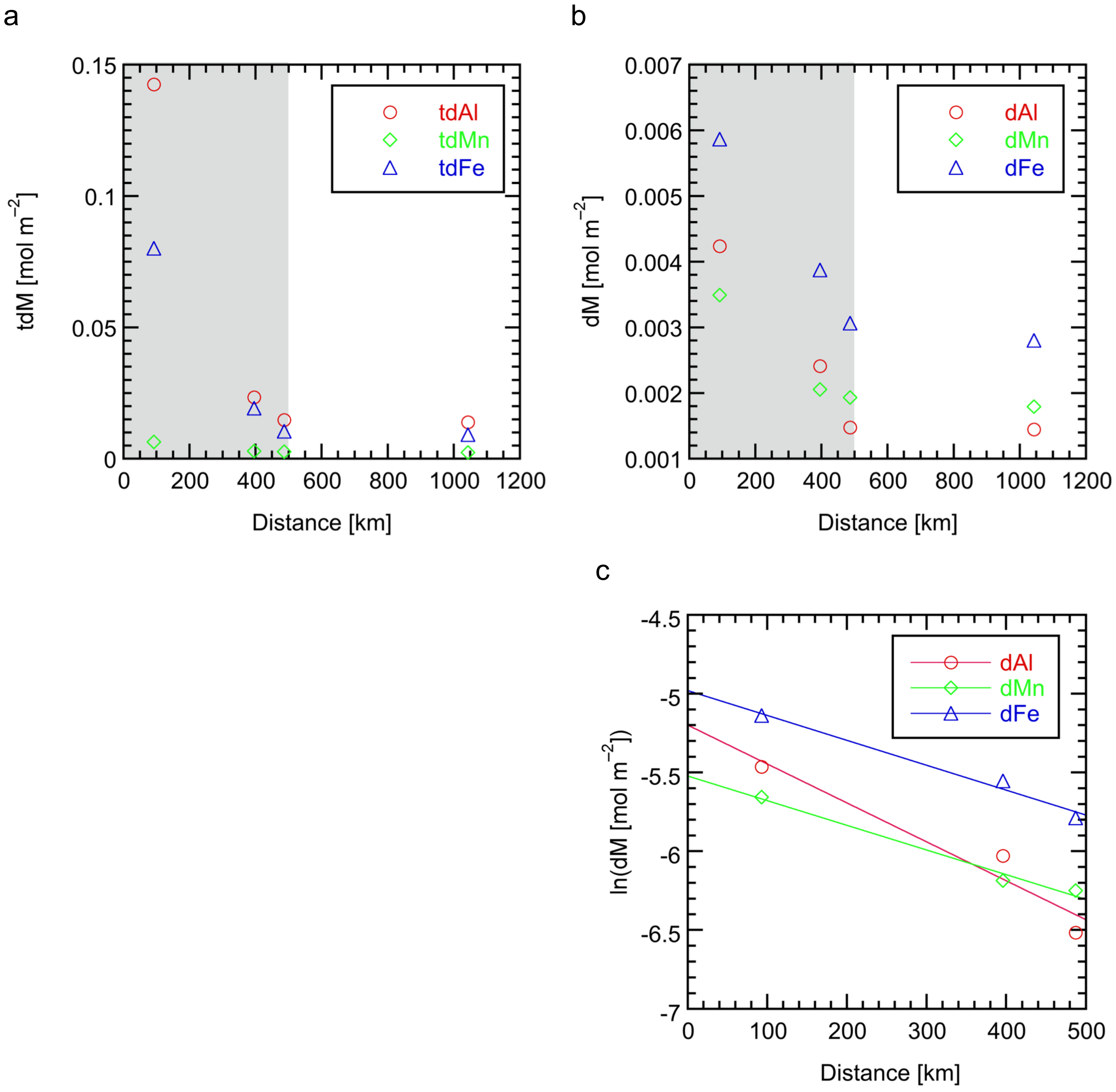

Supplementary Figure 14

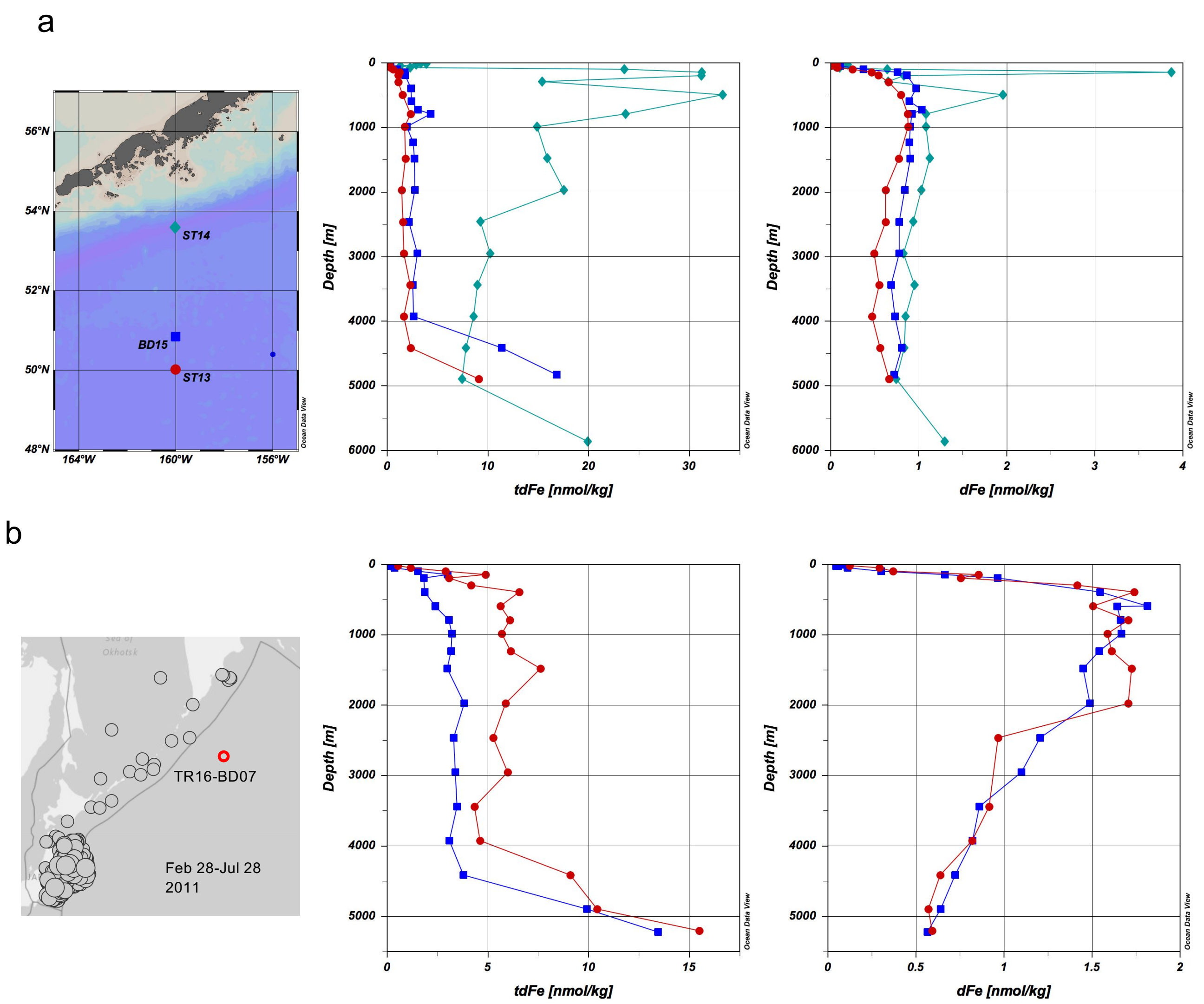

Supplementary Figure 15

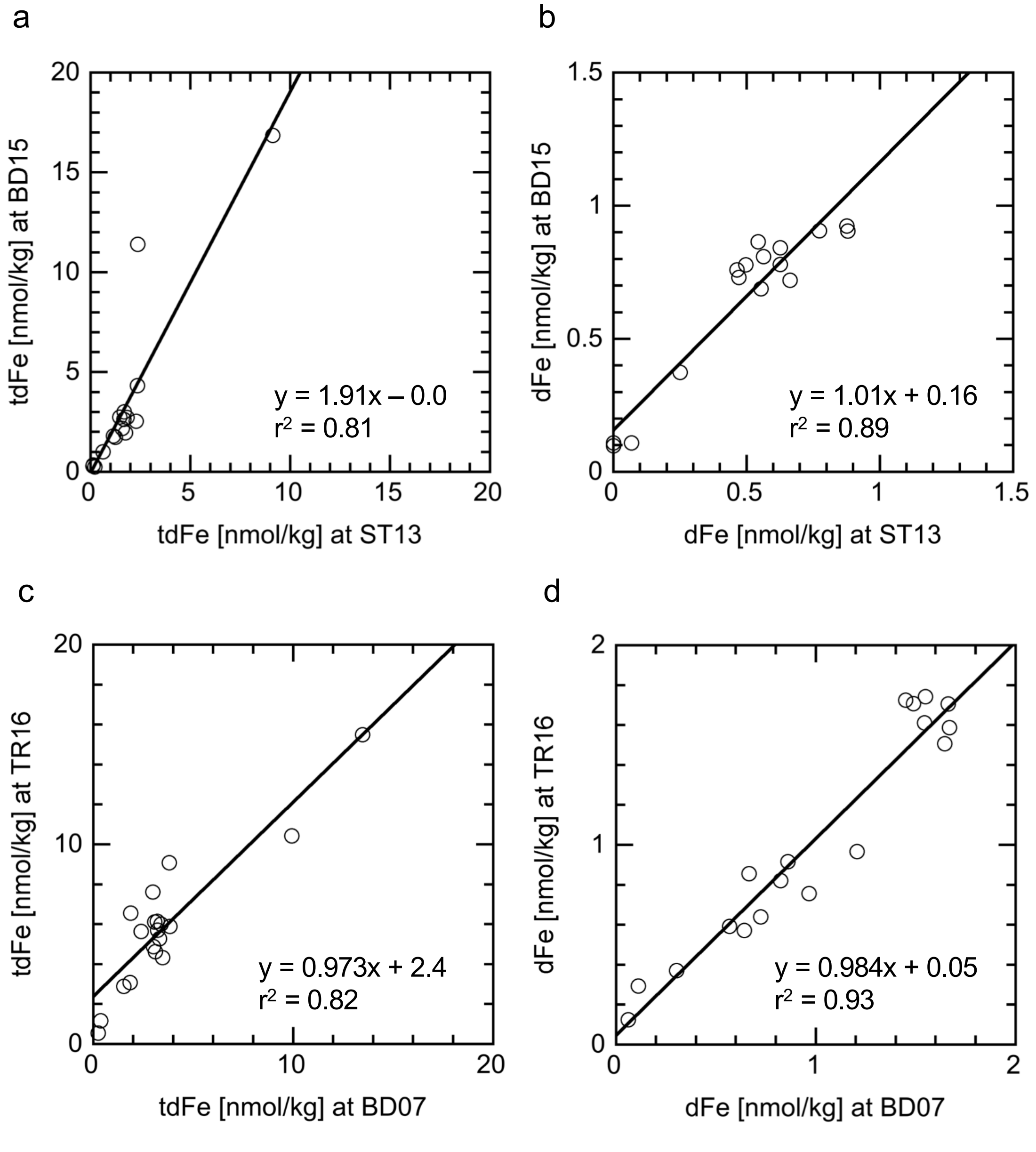

Supplementary Figure 16

Supplementary Table 1

(a separate file)

Supplementary Table 2 Statistical summary of seawater data

Element	fraction	*n*				minimum	maximum	median
		total	> DL	< DL	questionable	[nmol/kg]	[nmol/kg]	[nmol/kg]
Fe	td	638	630	2	6	0.05	33.3	1.43
	d	638	566	61	11	<0.04	3.87	0.55
	lp	638	601	20	17	<0.07	31.4	0.75

Supplementary Table 3 Summary of water mass data

Water mass	Salinity	Potential Temperature [°C]	σ_θ [kg/m^3]	Area	Reference	Fe [nmol/kg]		
						td	d	lp
Subtropical Mode Water (STMW)			25.2–25.8	30–44N, < 220E	Yasuda 2003	0.10-0.20	<0.04–0.11	<0.07–0.17
Central Mode Water (CMW)			26.0–26.7	30–44N, < 220E	Yasuda 2003	0.37–4.38	<0.04–0.57	0.22–3.96
Okhotsk Sea Mode Water (OSMW)	33.5–33.6	1–2	26.6–27	42–50N, < 166E	Yasuda 1997	1.83–4.89	0.46-0.97	0.87–4.03
North Pacific Intermediate Water (NPIW)	33.8–34.1		26.4–27.2, averaging 26.8	20–42N	Dickson et al. 2000, Bostock et al. 2010	0.60–3.21	0.17–0.87	0.44–2.64
Equatorial Pacific Intermediate Water (EqPIW)	34.5–34.6		27.3	< 20N	Bostock et al. 2010	0.83–1.52	0.43–0.75	0.35–1.02
Upper Circumpolar Deep Water (UCDW)	34.6	2.5	27.6	< 20N	Talley et al. 2012	0.86–1.85	0.42–0.69	0.39–1.43
Pacific Deep Water (PDW)	34.7	1.1–1.2	27.7	30–50.5N, 164–220E	Talley et al. 2011	0.89–3.32	0.38–1.28	0.34–2.51
Lower Circumpolar Deep Water (LCDW)	34.7	1–2	27.8		Talley et al. 2013	0.98–20.7	0.29–1.30	0.60–20.1

Supplementary Table 4. Inventory of tdM and dM in the North Pacific Ocean

	Area [km^2]	Inventory [mol]							
		tdAl	dAl	tdMn	dMn	tdFe	dFe	tdCo	dCo
Interior	6.47E+07	1.17E+12	2.70E+11	1.87E+11	1.14E+11	7.34E+11	2.22E+11	7.77E+09	6.77E+09
Boundary Scavenging Zone	1.23E+07	5.81E+11	4.28E+10	5.98E+10	3.74E+10	3.67E+11	5.70E+10	1.65E+09	1.38E+09
Total	7.70E+07	1.75E+12	3.12E+11	2.46E+11	1.51E+11	1.10E+12	2.78E+11	9.42E+09	8.15E+09

Supplementary Table 5 Procedure blanks and detection limits

Element	Fraction	KH-05-2 and KH-11-7			KH-12-4		
		Procedure blank		Detection limit	Procedure blank		Detection limit
		n	average		*n*	average	
Fe (nmol/kg)	td	16	0.02	0.03	20	0.03	0.06
	d	6	0.05	0.04			
	lp	-	-	0.07	-	-	0.10
